# Emergence of Multiple Novel Inter-Genotype Recombinant Strains of Human Astroviruses Detected in Pediatric Patients With Acute Gastroenteritis in Thailand

**DOI:** 10.3389/fmicb.2021.789636

**Published:** 2021-12-13

**Authors:** Hongyu Wei, Pattara Khamrin, Kattareeya Kumthip, Arpaporn Yodmeeklin, Niwat Maneekarn

**Affiliations:** ^1^Department of Microbiology, Faculty of Medicine, Chiang Mai University, Chiang Mai, Thailand; ^2^Department of Pathogenic Biology and Immunology, Youjiang Medical University for Nationalities, Baise, China; ^3^Center of Excellence in Emerging and Re-emerging Diarrheal Viruses, Chiang Mai University, Chiang Mai, Thailand

**Keywords:** human astrovirus, gastroenteritis, recombination, recombinant strain, pediatric patients, Thailand

## Abstract

**Objective:** Human astrovirus (HAstV) is recognized as an important cause of acute gastroenteritis in children. Recombination between different genotypes of HAstV can contribute to diversity and evolution of the virus. This study aimed to investigate the emergence of HAstV recombinant strains in pediatric patients hospitalized with acute gastroenteritis in Chiang Mai, Thailand, spanning 2011–2020.

**Methods:** A total of 92 archival HAstV strains collected from pediatric patients with acute gastroenteritis during 2011–2020 were further characterized to identify the recombinant strains. The ORF1b and ORF2 junction region of each strain was amplified and sequenced. The obtained sequences were analyzed in comparison with the reference sequences retrieved from GenBank database. Their genotypes were assigned using MEGA X software based on the partial ORF1b (RdRp) and ORF2 (capsid) regions, and the recombination breakpoints of recombinant strains were determined by SimPlot and RDP4 analyses.

**Results:** Five inter-genotype recombinant strains with three recombination patterns of ORF1b/ORF2 of classic HAstV, HAstV8/HAstV1, HAstV8/HAstV3, and HAstV3/HAstV2, were detected. The recombination breakpoints of all strains were located at the 3′-end region of ORF1b close to the ORF1b/ORF2 junction.

**Conclusion:** Several novel inter-genotype recombinant strains of classic HAstV genotypes were detected in pediatric patients with acute gastroenteritis in Chiang Mai, Thailand, during the period of 10 years from 2011 to 2020.

## Introduction

Human astrovirus (HAstV) is a non-enveloped, positive-sense, single-stranded RNA virus, which is associated with acute gastroenteritis and systemic diseases (Johnson et al., 2017). The genome of HAstV contains three open reading frames (ORFs), ORF1a, ORF1b, and ORF2 (Bosch et al., 2014). Currently, HAstVs are classified into three major groups, including classic HAstV (HAstV1-HAstV8), novel HAstV-MLB (MLB1-MLB3), and novel HAstV-VA/HMO (VA1-VA5) (De Benedictis et al., 2011; Bosch et al., 2014). The diversity of HAstV genotypes is plausibly associated with frequent interspecies transmission and recombination events (Wohlgemuth et al., 2019). The first evidence of recombination naturally occurring among HAstVs was documented in 2001 by demonstrating that the ORF1b and ORF2 of the new HAstV strain were closely related to those of HAstV3 and HAstV5, respectively, and a putative recombination site was demonstrated at the ORF1b/ORF2 junction (Walter et al., 2001). To date, many more recombinant strains have been reported even though little is known about the mechanism of astrovirus recombination (Bosch et al., 2014).

It is well documented that the recombination event is one of the mechanisms involved in the evolution of RNA viruses (Simon-Loriere and Holmes, 2011; Wohlgemuth et al., 2019). The epidemiology of HAstV has been studied in Thailand for decades (Malasao et al., 2012; Kumthip et al., 2018). However, no study on HAstV recombination has been carried out in Thailand. Therefore, the present study aimed to identify HAstV recombinant strains retrospectively in children hospitalized with acute gastroenteritis in Chiang Mai, Thailand, during the period of 2011–2020.

## Materials and Methods

### Fecal Samples and Human Astrovirus Strains

The fecal samples included in this study were collected from children under 5 years old who were admitted to five hospitals with acute gastroenteritis in Chiang Mai city, Thailand, during the period of 10 years from 2011 to 2020. The age of these in-patients ranged from neonate up to 5 years old. The criteria for acute gastroenteritis are defined as the sudden passage of watery stools (at least three times per day) for a duration of less than 2 weeks (Bassetto et al., 2019). A total of 3,534 fecal samples were collected and suspended in phosphate-buffered saline (PBS), pH 7.4, for viral genome extraction and screened for HAstV by the RT-PCR method using panastrovirus consensus primers (Finkbeiner et al., 2009a). Out of 3,534 fecal samples, 92 were positive for HAstV, and their genotypes, based on the ORF1b (RdRp) nucleotide sequence analysis, were assigned by comparison with those of the corresponding reference strains available in the GenBank database by performing the nucleotide sequence alignment and phylogenetic analyses as described previously (Kumthip et al., 2018). The ORF1b (RdRp) nucleotide sequences of these 92 HAstV strains have been deposited in the GenBank database under the access numbers of MH325213-MH325266, and MZ327095-MZ327133. Of 92 HAstV strains, 70 were classic HAstV; 19 and 3 were novel HAstV-MLB and novel HAstV-VA, respectively; and all of these HAstV strains were included in this study. More comprehensive information about the epidemiology, prevalence, seasonality, genotype distribution, and coinfection of HAstV with other diarrheal viruses of the stool samples included in this study are available in our published articles (Kumthip et al., 2018; Wei et al., 2021).

### Amplification and Nucleotide Sequencing of Human Astrovirus ORF1b (RdRp) and ORF2 (capsid) Junction

The viral RNA genomes of 92 archival HAstV strains were extracted using a Geneaid Viral Nucleic Acid Extraction Kit II (Geneaid, Taipei, Taiwan) according to the manufacturer’s protocol. The cDNA was synthesized from the viral RNA genome using RevertAid Fist Stand cDNA Synthesis Kit (Thermo Fisher Scientific, United States). Amplification of the ORF1b and ORF2 junction encompassing the 3′ end of ORF1b and 5′ end of ORF2 was performed by PCR and semi-nested PCR using the primer sets listed in Table 1. For classic HAstV, a semi-nested PCR was performed using two different alternative forward primers, SF0073 and SF0076-F, in combination with a set of outer reverse primer AHAstVR1 and inner reverse primer AHAstVR2, which generated PCR product sizes of 1104 and 708 bp, respectively. For novel HAstV-MLB, a conventional PCR was performed using forward primer SF0073 and reverse primer AHMLBR1 to generate a PCR product size of 926 bp. For novel HAstV-VA, a semi-nested PCR was performed using forward primer SF0076-F in combination with a set of outer reverse primer AHVAR1 and inner reverse primer AHVAR2, which generated a PCR product size of 987 bp. The PCR products were purified by using the GenepHlow™ Gel/PCR kit (Geneaid Biotech, Taiwan), and then, the purified PCR products were sequenced by First Base Laboratory SDNBHN Selangor Darul Ehsan, Malaysia.

**TABLE 1:** Primers used for PCR and semi-nested PCR to amplify the ORF1b and ORF2 junction.

Target	Assay	Primer name (direction)	Sequence (5′→ 3′)	Location	PCR product size	References
Classic HAstV	Semi-nested PCR (1st and 2nd round)	SF0073 (forward)*^a^*	GAYTGGACWCGATTTGATGGTAC	3583–3605	–	Finkbeiner et al., 2009b; Hata et al., 2015
	Semi-nested PCR (1st and 2nd round)	SF0076-F (forward)*^b^*	GGAATGTGGGTTAAGCCAG	3979–3997	–	This study
	Semi-nested PCR (1st round)	AHAstVR1 (reverse)	CCTARCGCYTGCACDGG	4697–4713	1131 bp*^a^* 735 bp*^b^*	Hata et al., 2014
	Semi-nested PCR (2nd round)	AHAstVR2 (reverse)	GTRCTYCCWGTAGCRTCCTTAAC	4664–4686	1104 bp*^a^* 708 bp*^b^*	Hata et al., 2014
Novel HAstV-MLB	PCR	SF0073 (forward)	GAYTGGACWCGATTTGATGGTAC	3110–3132	–	Finkbeiner et al., 2009b; Hata et al., 2015
	PCR	AHMLBR1 (reverse)	CAGGYTTAGGCCCAGTTGTA	4016–4035	926 bp	Hata et al., 2015
Novel HAstV-VA	Semi-nested PCR (1st and 2nd round)	SF0076-F (forward)	GGAATGTGGGTTAAGCCAG	3855–3873	–	This study
	Semi-nested PCR (1st round)	AHVAR1 (reverse)	ARTTTCTTGACAAACCACCAWCC	5224–5246	1392 bp	Hata et al., 2015
	Semi-nested PCR (2nd round)	AHVAR2 (reverse)	SCTCCCTCTTCATTKGTRTCTGT	4819–4841	987 bp	Hata et al., 2015

*^a^Primary primer for classic HAstV.*

*^b^Optional primer for classic HAstV.*

*The mixed bases in the degenerate primer are as follows: Y, for C or T; W, for A or T; R, for A or G; D, for A, G, or T; S, for G or C; K, for T or G. The primer locations for classic HAstV, novel HAstV-MLB, and novel HAstV-VA strains were based on the nucleotide sequence positions of L23513, FJ402983, and GQ502193 reference strains from GenBank database, respectively.*

### Phylogenetic and Recombination Analyses

The obtained nucleotide sequences were analyzed by comparing with those of the reference strains available in the GenBank database using the Basic Local Alignment Search Tool (BLAST) server.^1^ The phylogenetic trees of the partial ORF1b (RdRp) and ORF2 (capsid) genes were constructed by using the MEGA X software (Tamura, 1992; Kumar et al., 2018) based on the maximum likelihood method and selected the best-fit evolutionary model for the data set *via* Tamura-3-parameter model with 1,000 replicates. The recombination breakpoint was determined by using SimPlot software v.3.5.1 (Lole et al., 1999) and Recombination Detection Program v.4.39 (RDP 4.39) (Wang et al., 2020). In addition, identification of the homologous position of the recombination breakpoint was also based on nucleotide sequence alignment of our strains together with other reference strains.

### Nucleotide Sequence Accession Number

The nucleotide sequences of HAstV recombinant strains described in this study have been deposited in the GenBank database under the accession numbers OK135150-OK135154.

## Results

### Phylogenetic Analysis and Recombination Patterns of Putative Recombinant Strains

**FIGURE 1:**
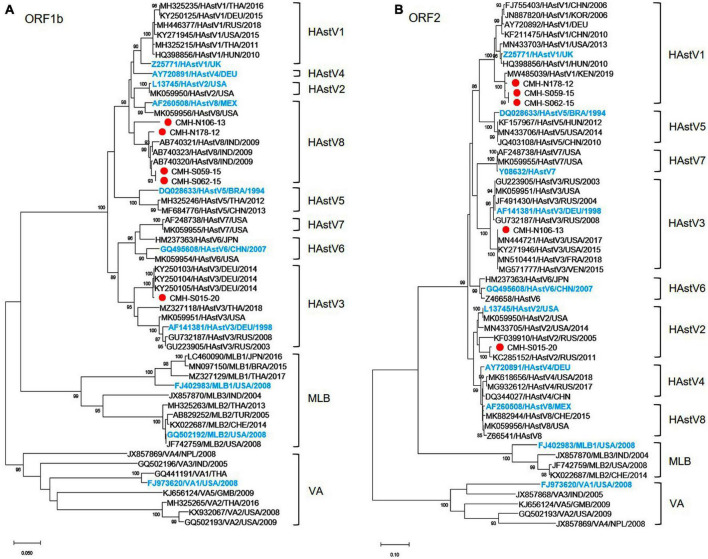
Phylogenetic analysis of classic HAstV recombinant strains based on **(A)** partial nucleotide sequence of ORF1b (RdRp) region and **(B)** partial nucleotide sequence of ORF2 (capsid) region. The putative recombinant strains detected in this study are indicated by a red solid circle. The International Committee on Taxonomy of Viruses (ICTV) approved reference strains are highlighted in blue.

Analysis of the nucleotide sequences of partial ORF1b (RdRp) (342 bp) and ORF2 (capsid) (329 bp) of 92 HAstV strains detected in this study revealed that assigned genotypes based on ORF1b of only five strains (CMH-N178-12, CMH-N106-13, CMH-S059-15, CMH-S062-15, and CMH-S015-20) did not coincide with the genotypes based on ORF2 region. The data implied that these five strains of classic HAstV were putative recombinant strains. The recombination evidence of these strains was confirmed by further phylogenetic analysis using the nucleotide sequences of ORF1b and ORF2 of these strains as shown in Figures 1A,B, respectively. The phylogenetic tree of ORF1b (Figure 1A) showed that four strains of HAstV (CMH-N178-12, CMH-N106-13, CMH-S059-15, and CMH-S062-15) are clustered closely together with the classic HAstV8 reference strains reported previously from Mexico (AF260508), the United States (MK059956), and India (AB740320, AB740321, and AB740323) with the nucleotide sequence identities ranging from 90.2–99.0%. The data indicate that the ORF1b of CMH-N178-12, CMH-N106-13, CMH-S059-15, and CMH-S062-15 are classic HAstV8 genotypes. However, the phylogenetic tree of ORF2 (Figure 1B) of CMH-N178-12, CMH-N106-13, CMH-S059-15, and CMH-S062-15 shows that CMH-N178-12, CMH-S059-15, and CMH-S062-15 clustered together with classic HAstV1 reference strains reported previously from the United Kingdom (Z25771), China (FJ755403 and KF211475), Korea (JN887820), Germany (AY720892), Hungary (HQ398856), the United States (MN433703), and Kenya (MW485039) with the nucleotide sequence identities ranging from 91.9–98.1%, whereas CMH-N106-13 clustered together with the HAstV3 reference strains reported previously from Germany (AF141381), the United States (MN444721, MK059951, and KY271946), France (MN510441), Venezuela (MG571777), and Russia (JF491430, GU732187, and GU223905) with the nucleotide sequence identities ranging from 95.7–100%. The data suggested that the genotype of CMH-N178-12, CMH-S059-15, and CMH-S062-15 based on the ORF2 region, were HAstV1, whereas that of CMH-N106-13 was a HAstV3 genotype. Altogether, the data indicate that CMH-N178-12, CMH-S059-15, and CMH-S062-15 were the inter-genotype recombinant strains of classic HAstV8 and HAstV1 genotypes, whereas CMH-N106-13 was an inter-genotype recombinant strain of classic HAstV8 and HAstV3 genotypes. In addition, one more putative inter-genotype recombinant strain, CMH-S015-20, was detected in this study. The phylogenetic tree of nucleotide sequence of ORF1b of CMH-S015-20 (Figure 1A) revealed that CMH-S015-20 clustered closely together with the classic HAstV3 reference strains reported previously from Germany (AF141381, KY250103, KY250104, and KY250105), Thailand (MZ327118), the United States (MK059951), and Russia (GU223905 and GU732187) with the nucleotide sequence identities ranging from 90.7–99.6%. The data suggest that the ORF1b of CMH-S015-20 is a HAstV3 genotype. Nevertheless, the phylogenetic tree of nucleotide sequence of ORF2 of CMH-S015-20 (Figure 1B) shows that CMH-S015-20 clustered closely together with the classic HAstV2 reference strains reported previously from Russia (KF039910 and KC285152) and the United States (MN433705, L13745, and MK059950) with the nucleotide sequence identities ranging from 91–98.4%. The data suggest that the ORF2 of CMH-S015-20 is a HAstV2 genotype. Altogether, CMH-S015-20 contains the ORF1b of HAstV3 and ORF2 of HAstV2 genotypes, indicating that CMH-S015-20 strain is an inter-genotype recombinant of HAstV3 and HAstV2 genotypes.

### Analysis of Recombination Breakpoints

**FIGURE 2:**
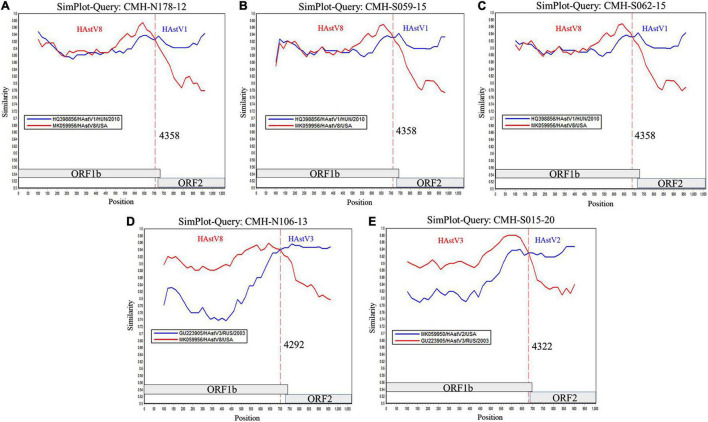
Similarity plot for HAstV recombinant strains detected in Chiang Mai, Thailand, 2011–2020. Representative sequences from each of five recombinant strains (ORF1b/ORF2) detected in this study (query strains) are **(A)** CMH-N178-12 (HAstV8/HAstV1), **(B)** CMH-S059-15 (HAstV8/HAstV1), **(C)** CMH-S062-15 (HAstV8/HAstV1), **(D)** CMH-N106-13 (HAstV8/HAstV3), and **(E)** CMH-S015-20 (HAstV3/HAstV2). The line intersection defines the predicted recombination site (vertical dashed line). The *x*-axis represents the nucleotide position, and the *y*-axis indicates the similarity between the query strain and the reference strains.

To locate the potential recombination breakpoints within the ORF1b and ORF2 junction region of the putative inter-genotype recombinant strains, the SimPlot software and Recombination Detection Program (RDP4) were used. For the classic HAstV8/HAstV1 recombination pattern, the nucleotide sequences of the ORF1b and ORF2 junction of CMH-N178-12, CMH-S059-15, and CMH-S062-15 were analyzed in comparison with those of the classic HAstV8 (MK059956/HAstV8/USA) and classic HAstV1 (HQ398856/HAstV1/HUN/2010) reference strains (Figure 2). The recombination breakpoints of CMH-N178-12 (Figure 2A), CMH-S059-15 (Figure 2B), and CMH-S062-15 (Figure 2C) are located at the same nucleotide (nt) position 4358 based on the genome nt position of classic HAstV1 reference strain (HQ398856/HAstV1/HUN/2010). The recombination breakpoint located at the 3′-end region of ORF1b closed to ORF1b/ORF2 junction, with maximum chi-square value of 1.508 × 10^–3^, 9.415 × 10^–5^, and 1.945 × 10^–5^, respectively (Table 2).

**TABLE 2:** Recombination patterns and characteristics of the classic HAstV recombinant strains detected in Chiang Mai, Thailand, 2011–2020.

Year	Strain name	HAstV genotype		Prototypes used for SimPlot analysis	Predicted Recombination point (nt position)	Recombination region in ORF	Maximum chi-square value
		ORF1b	ORF2				
2012	CMH-N178-12	HAstV8	HAstV1	HAstV8 (MK059956) HAstV1 (HQ398856)	4358	ORF1b	1.508 × 10^–3^
2015	CMH-S059-15	HAstV8	HAstV1	HAstV8 (MK059956) HAstV1 (HQ398856)	4358	ORF1b	9.415 × 10^–5^
2015	CMH-S062-15	HAstV8	HAstV1	HAstV8 (MK059956) HAstV1 (HQ398856)	4358	ORF1b	1.945 × 10^–5^
2013	CMH-N106-13	HAstV8	HAstV3	HAstV8 (MK059956) HAstV3 (GU223905)	4292	ORF1b	4.293 × 10^–11^
2020	CMH-S015-20	HAstV3	HAstV2	HAstV3 (GU223905) HAstV2 (MK059950)	4322	ORF1b	2.414 × 10^–6^

For a classic HAstV8/HAstV3 recombination pattern, the nucleotide sequence of ORF1b and ORF2 junction of CMH-N106-13 was analyzed in comparison with those of the classic HAstV8 (MK059956/HAstV8/USA) and classic HAstV3 (GU223905/HAstV3/RUS/2003) reference strains. The recombination breakpoint of CMH-N106-13 was observed at nt position 4292 based on the genome nt position of the classic HAstV3 reference strain (GU223905/HAstV3/RUS/2003), which is located at the 3′-end region of ORF1b close to the ORF1b/ORF2 junction (Figure 2D) with a maximum chi-square value of 4.293 × 10^–11^ (Table 2).

For the classic HAstV3/HAstV2 recombination pattern, the nucleotide sequence of the ORF1b and ORF2 junction of CMH-S015-20 was analyzed in comparison with those of the classic HAstV3 (GU223905/HAstV3/RUS/2003) and classic HAstV2 (MK059950/HAstV2/USA) reference strains. The recombination breakpoint was observed at nt position 4322 based on the genome nt position of the classic HAstV2 reference strain (MK059950/HAstV2/USA), which is located at the 3′-end region of ORF1b close to ORF1b/ORF2 junction (Figure 2E) with maximum chi-square value of 2.414 × 10^–6^ (Table 2).

## Discussion

The genetic recombination is commonly found in the RNA virus families (Lai, 1992; Worobey and Holmes, 1999). As with most other RNA viruses, HAstV recombination was initially reported for the first time in 2001 by Walter et al. (2001), and later, recombination was proposed as one of the mechanisms that plays a crucial role in the genetic diversity and evolution of HAstV (Wohlgemuth et al., 2019; Roach and Langlois, 2021). Since then, a number of studies have reported a wide variety of recombinant strains, and the ORF1b/ORF2 junction of the HAstV genome has been reported as the predominant location at which recombination events occur (Walter et al., 2001; Wolfaardt et al., 2011; De Grazia et al., 2012; Martella et al., 2013; Babkin et al., 2014; Wohlgemuth et al., 2019). So far, several inter-genotypes of the ORF1b/ORF2 recombinant strains have been reported from many countries around the world. The recombination at the ORF1b/ORF2 region of HAstV3/HAstV5 was first reported from the United States and Mexico in 2001 (Walter et al., 2001). The recombinations at the ORF1b/ORF2 region of HAstV1/HAstV2, HAstV3/HAstV2, HAstV1/HAstV3, and HAstV1/HAstV4 were reported from Italy (De Grazia et al., 2012; Martella et al., 2013). In addition to Italy, the HAstV3/HAstV2 was also reported from Russia (Babkin et al., 2014) and Kenya (Wolfaardt et al., 2011). The HAstV4/HAstV5 was reported from South Africa (Taylor et al., 2001) and China (Zhou et al., 2016). In addition, the HAstV1/HAstV5, HAstV2/HAstV5, and HAstV1/HAstV2 were also reported from China (Zhou et al., 2016). Furthermore, the HAstV2/HAstV8 was reported from South Korea (Ha et al., 2016).

Our study reports the detection of five isolates of ORF1b/ORF2 recombinant strains of HAstV8/HAstV1 (*n* = 3), HAstV8/HAstV3 (*n* = 1), and HAstV3/HAstV2 (*n* = 1) in children with acute gastroenteritis (Figure 2). The HAstV8/HAstV1 was detected in three out of five (60.0%) of the ORF1b/ORF2 recombinant strains described in this study, suggesting that a recombination event between HAstV8 and HAstV1 likely occurs more commonly than between other genotypes. Moreover, it is interesting to point out that the recombinant strains of HAstV8/HAstV1 and HAstV8/HAstV3 at the ORF1b/ORF2 region, to the best of our knowledge, have not been reported previously from elsewhere and are being reported here in this study as the novel HAstV ORF1b/ORF2 recombinant strains. Nevertheless, the HAstV8/HAstV1 recombinant strain at the ORF1a/ORF2 region has been reported previously from India with high predominance of 67.7–76.9% (Verma et al., 2010; Pativada et al., 2011) compared to other inter-genotype recombinant strains detected in the same study. In the present study, the HAstV8/HAstV1 recombinant strains were detected in 2012 and 2015, whereas HAstV8/HAstV3 was detected in 2013 in Chiang Mai, Thailand (Table 2). To ascertain that the recombination events had occurred in nature to generate these recombinant strains, HAstV8, HAstV1, and HAstV3 should have been demonstrated to circulate in this geographical area prior to or at the same period of time when the recombinant strains were detected. In fact, HAstV8 and HAstV1 were reported previously in children with acute gastroenteritis in this geographical area during the period of 2011–2016 (Kumthip et al., 2018) and HAstV3 in 2000 (Malasao et al., 2012).

The ORF1b/ORF2 region of the HAstV genome is demonstrated to be a predominant location at which recombination events frequently occur (Walter et al., 2001; Wolfaardt et al., 2011; De Grazia et al., 2012; Martella et al., 2013; Babkin et al., 2014; Wohlgemuth et al., 2019). In our study, the recombination breakpoints of all five recombinant strains were located close to the 3′-end region of ORF1b (Figure 2). All three novel HAstV8/HAstV1 recombinant strains (CMH-N178-12, CMH-S059-15, and CMH-S062-15) contained recombination breakpoint at the same nt position 4358, suggesting that nt position 4358 is a hot spot for recombination events to occur between HAstV8 and HAstV1 genotypes. In addition, the recombination breakpoint of novel HAstV8/HAstV3 (CMH-N106-13) recombinant strain located at nt position 4292 of ORF1b region. Regarding the recombination breakpoint of CMH-S015-20 located at nt position 4322 of ORF1b, HAstV2 (MK059950/HAstV2/USA) was used as the reference strain, whereas the HAstV3/HAstV2 recombinant strain reported from Kenya (Wolfaardt et al., 2011) used HAstV1 (L23513/HAstV1/USA) as a reference strain to predict the recombination breakpoint at nt position 4328. We performed a nucleotide sequence alignment of these three reference strains and found the recombination breakpoint located at nt positions 4322, 4248, and 4325 based on HAstV2, HAstV3, HAstV1 as the reference strains, respectively (data not shown). Even though the recombination breakpoints are different between different HAstV genotypes, in fact, the recombination breakpoint occurs at the same nt position. The difference in the number of the nt position is based on the nt position in the full-length genome of each HAstV genotype used as the reference strain.

Besides the recombination events being shown in the classic HAstV, suggestive evidence of recombination between novel HAstV-MLB3 and HAstV-MLB1 or -MLB2 by using next generation sequencing the samples from environmental waters has been reported recently (Hata et al., 2018). Since then, no other reports are available in the literature. In the present study, we also investigated the recombinant strains among HAstV-MLB and HAstV-VA strains; no recombination event has been identified in this study. To date, no recombination of classic HAstV with either HAstV-MLB or HAstV-VA has been reported.

## Data Availability Statement

The data presented in the study are deposited in the GenBank database, accession number OK135150–OK135154 (https://www.ncbi.nlm.nih.gov/genbank/).

## Ethics Statement

The studies involving human participants were reviewed and approved by the ethical committee for human rights related to human experimentation, Faculty of Medicine, Chiang Mai University (MIC-2557-02710). Written informed consent to participate in this study was provided by the participants’ legal guardian/next of kin.

## Author Contributions

HW performed the experiments, data collection and analysis, drafted the original and made a revised manuscript, and read and approved the final draft. PK and KK conceived and designed the study, funding acquisition, specimen collection, supervised the study procedures, data collection and analysis, and read and approved the final draft manuscript. AY supervised the study procedures, and performed the experiments, and read and approved the final draft manuscript. NM conceived and designed the study, funding acquisition, analyzed and interpreted the data, was responsible for the overall study, and critically revised and editing the manuscript. All authors contributed to the article and approved the submitted version.

## Conflict of Interest

The authors declare that the research was conducted in the absence of any commercial or financial relationships that could be construed as a potential conflict of interest.

## Publisher’s Note

All claims expressed in this article are solely those of the authors and do not necessarily represent those of their affiliated organizations, or those of the publisher, the editors and the reviewers. Any product that may be evaluated in this article, or claim that may be made by its manufacturer, is not guaranteed or endorsed by the publisher.

## Footnotes

1
https://blast.ncbi.nlm.nih.gov/Blast.cgi

## References

[B1] Babkin I. V. Tikunov A. Y. Sedelnikova D. A. Zhirakovskaia E. V. Tikunova N. V. (2014). Recombination analysis based on the HAstV-2 and HAstV-4 complete genomes. *Infect. Genet. Evol.* 22 94–102. 10.1016/j.meegid.2014.01.010 24462746

[B2] Bassetto M. Van Dycke J. Neyts J. Brancale A. Rocha-Pereira J. (2019). Targeting the viral polymerase of diarrhea-causing viruses as a strategy to develop a single broad-spectrum antiviral therapy. *Viruses* 11:173. 10.3390/v11020173 30791582 PMC6409847

[B3] Bosch A. Pintó R. M. Guix S. (2014). Human astroviruses. *Clin. Microbiol. Rev.* 27 1048–1074. 10.1128/CMR.00013-14 25278582 PMC4187635

[B4] De Benedictis P. Schultz-Cherry S. Burnham A. Cattoli G. (2011). Astrovirus infections in humans and animals–molecular biology, genetic diversity, and interspecies transmissions. *Infect. Genet. Evol.* 11 1529–1544. 10.1016/j.meegid.2011.07.024 21843659 PMC7185765

[B5] De Grazia S. Medici M. Pinto P. Moschidou P. Tummolo F. Calderaro A. (2012). Genetic heterogeneity and recombination in human type 2 astroviruses. *J. Clin. Microbiol.* 50 3760–3764. 10.1128/JCM.02102-12 22933603 PMC3486218

[B6] Finkbeiner S. R. Holtz L. R. Jiang Y. Rajendran P. Franz C. J. Zhao G. (2009a). Human stool contains a previously unrecognized diversity of novel astroviruses. *Virol. J.* 6:161. 10.1186/1743-422X-6-161 19814825 PMC2765957

[B7] Finkbeiner S. R. Le B. M. Holtz L. R. Storch G. A. Wang D. (2009b). Detection of newly described astrovirus MLB1 in stool samples from children. *Emerg. Infect. Dis.* 15 441–444. 10.3201/eid1503.081213 19239759 PMC2666294

[B8] Ha H. J. Lee S. G. Cho H. G. Jin J. Y. Lee J. W. Paik S. Y. (2016). Complete genome sequencing of a recombinant strain between human astrovirus antigen types 2 and 8 isolated from South Korea. *Infect. Genet. Evol.* 39 127–131. 10.1016/j.meegid.2016.01.017 26812127

[B9] Hata A. Katayama H. Kitajima M. Furumai H. (2015). Wastewater analysis indicates that genetically diverse astroviruses, including strains belonging to novel clades MLB and VA, are circulating within Japanese populations. *Appl. Environ. Microbiol.* 81 4932–4939. 10.1128/AEM.00563-15 25979884 PMC4495221

[B10] Hata A. Kitajima M. Haramoto E. Lee S. Ihara M. Gerba C. P. (2018). Next-generation amplicon sequencing identifies genetically diverse human astroviruses, including recombinant strains, in environmental waters. *Sci. Rep.* 8 1–9. 10.1038/s41598-018-30217-y 30087387 PMC6081416

[B11] Hata A. Kitajima M. Tajiri-Utagawa E. Katayama H. (2014). Development of a high resolution melting analysis for detection and differentiation of human astroviruses. *J. Virol. Methods* 200 29–34. 10.1016/j.jviromet.2014.01.023 24509176

[B12] Johnson C. Hargest V. Cortez V. Meliopoulos V. A. Schultz-Cherry S. (2017). Astrovirus Pathogenesis. *Viruses* 9:22. 10.3390/v9010022 28117758 PMC5294991

[B13] Kumar S. Stecher G. Li M. Knyaz C. Tamura K. (2018). MEGA X: molecular evolutionary genetics analysis across computing platforms. *Mol. Biol. Evol.* 35 1547–1549. 10.1093/molbev/msy096 29722887 PMC5967553

[B14] Kumthip K. Khamrin P. Ushijima H. Maneekarn N. (2018). Molecular epidemiology of classic, MLB and VA astroviruses isolated from <5 year-old children with gastroenteritis in Thailand, 2011-2016. *Infect. Genet. Evol.* 65 373–379. 10.1016/j.meegid.2018.08.024 30153477

[B15] Lai M. (1992). RNA recombination in animal and plant viruses. *Microbiol. Mol. Biol. Rev.* 56 61–79. 10.1128/mr.56.1.61-79.1992 1579113 PMC372854

[B16] Lole K. S. Bollinger R. C. Paranjape R. S. Gadkari D. Kulkarni S. S. Novak N. G. (1999). Full-length human immunodeficiency virus type 1 genomes from subtype C-infected seroconverters in India, with evidence of intersubtype recombination. *J. Virol.* 73 152–160. 10.1128/JVI.73.1.152-160.1999 9847317 PMC103818

[B17] Malasao R. Khamrin P. Chaimongkol N. Ushijima H. Maneekarn N. (2012). Diversity of human astrovirus genotypes circulating in children with acute gastroenteritis in Thailand during 2000-2011. *J. Med. Virol.* 84 1751–1756. 10.1002/jmv.23396 22997078

[B18] Martella V. Medici M. C. Terio V. Catella C. Bozzo G. Tummolo F. (2013). Lineage diversification and recombination in type-4 human astroviruses. *Infect. Genet. Evol.* 20 330–335. 10.1016/j.meegid.2013.09.015 24084291

[B19] Pativada M. S. Chatterjee D. Mariyappa N. S. Rajendran K. Bhattacharya M. K. Ghosh M. (2011). Emergence of unique variants and inter-genotype recombinants of human astroviruses infecting infants, children and adults in Kolkata, India. *Int. J. Mol. Epidemiol. Genet.* 2 228–235. 21915361 PMC3166150

[B20] Roach S. N. Langlois R. A. (2021). Intra- and cross-species transmission of astroviruses. *Viruses* 13:1127. 10.3390/v13061127 34208242 PMC8230745

[B21] Simon-Loriere E. Holmes E. C. (2011). Why do RNA viruses recombine? *Nat. Rev. Microbiol.* 9 617–626. 10.1038/nrmicro2614 21725337 PMC3324781

[B22] Tamura K. (1992). Estimation of the number of nucleotide substitutions when there are strong transition-transversion and G+C-content biases. *Mol. Biol. Evol.* 9 678–687. 10.1093/oxfordjournals.molbev.a040752 1630306

[B23] Taylor M. B. Walter J. Berke T. Cubitt W. D. Mitchell D. K. Matson D. O. (2001). Characterisation of a South African human astrovirus as type 8 by antigenic and genetic analyses. *J. Med. Virol.* 64 256–261. 10.1002/jmv.1044 11424112

[B24] Verma H. Chitambar S. D. Gopalkrishna V. (2010). Astrovirus associated acute gastroenteritis in western India: predominance of dual serotype strains. *Infect. Genet. Evol.* 10 575–579. 10.1016/j.meegid.2010.01.008 20117249

[B25] Walter J. Briggs J. Guerrero M. Matson D. Pickering L. Ruiz-Palacios G. (2001). Molecular characterization of a novel recombinant strain of human astrovirus associated with gastroenteritis in children. *Arch. Virol.* 146 2357–2367. 10.1007/s007050170008 11811685 PMC7087139

[B26] Wang Y. Guo X. Cui Y. Zhou Y. Yang K. Fu Z. (2020). Genetic characterization and phylogenetic analysis of feline astrovirus from Anhui province in eastern China. *3 Biotech* 10:354. 10.1007/s13205-020-02308-z 32766095 PMC7385053

[B27] Wei H. Khamrin P. Kumthip K. Yodmeeklin A. Maneekarn N. (2021). High divergence of human astrovirus genotypes circulating in pediatric patients hospitalized with acute gastroenteritis in Chiang Mai, Thailand, 2017–2020. *Sci. Rep.* 11:23266. 10.1038/s41598-021-02745-7 34853390 PMC8636499

[B28] Wohlgemuth N. Honce R. Schultz-Cherry S. (2019). Astrovirus evolution and emergence. *Infect. Genet. Evol.* 69 30–37. 10.1016/j.meegid.2019.01.009 30639546 PMC7106029

[B29] Wolfaardt M. Kiulia N. M. Mwenda J. M. Taylor M. B. (2011). Evidence of a recombinant wild-type human astrovirus strain from a Kenyan child with gastroenteritis. *J. Clin. Microbiol.* 49 728–731. 10.1128/jcm.01093-10 21106800 PMC3043485

[B30] Worobey M. Holmes E. C. (1999). Evolutionary aspects of recombination in RNA viruses. *J. Gen. Virol.* 80 2535–2543. 10.1099/0022-1317-80-10-2535 10573145

[B31] Zhou N. Lin X. Wang S. Wang H. Bi Z. Wang P. (2016). Molecular characterization of classic human astrovirus in eastern China, as revealed by environmental sewage surveillance. *J. Appl. Microbiol.* 120 1436–1444. 10.1111/jam.13109 26913699

